# Abundance of Indo-Pacific bottlenose dolphins (*Tursiops aduncus*) along the south coast of South Africa

**DOI:** 10.1371/journal.pone.0227085

**Published:** 2020-10-12

**Authors:** O. Alejandra Vargas-Fonseca, Stephen P. Kirkman, W. Chris Oosthuizen, Thibaut Bouveroux, Vic Cockcroft, Danielle S. Conry, Pierre A. Pistorius

**Affiliations:** 1 Department of Zoology, Nelson Mandela University, Port Elizabeth, South Africa; 2 Marine Apex Predator Research Unit (MAPRU), Institute for Coastal and Marine Research, Nelson Mandela University, Port Elizabeth, South Africa; 3 Branch: Oceans and Coasts, Department of Environment, Forestry and Fisheries (DEFF), Cape Town, South Africa; 4 Department of Zoology and Entomology, Mammal Research Institute, University of Pretoria, Hatfield, South Africa; Institute of Deep-sea Science and Engineering, Chinese Academy of Sciences, CHINA

## Abstract

Coastally distributed dolphin species are vulnerable to a variety of anthropogenic pressures, yet a lack of abundance data often prevents data-driven conservation management strategies from being implemented. We investigated the abundance of Indo-Pacific bottlenose dolphins (*Tursiops aduncus*) along the south coast of South Africa, from the Goukamma Marine Protected Area (MPA) to the Tsitsikamma MPA, between 2014 and 2016. During this period, 662.3h of boat-based photo-identification survey effort was carried out during 189 surveys. The sighting histories of 817 identified individuals were used to estimate abundance using capture-recapture modelling. Using open population (POPAN) models, we estimated that 2,155 individuals (95% CI: 1,873–2,479) occurred in the study area, although many individuals appeared to be transients. We recorded smaller group sizes and an apparent decline in abundance in a subset of the study area (Plettenberg Bay) compared to estimates obtained in 2002–2003 at this location. We recorded declines of more than 70% in both abundance and group size for a subset of the study area (Plettenberg Bay), in relation to estimates obtained in 2002–2003 at this location. We discuss plausible hypotheses for causes of the declines, including anthropogenic pressure, ecosystem change, and methodological inconsistencies. Our study highlights the importance of assessing trends in abundance at other locations to inform data-driven conservation management strategies of *T*. *aduncus* in South Africa.

## Introduction

Information on the abundance and trends of wildlife populations is essential to inform species and ecosystem conservation management strategies [1, 2]. Abundance trends indicate natural or anthropogenic driven ecosystem changes and can provide evidence on the efficacy of implemented conservation strategies [3]. In both terrestrial and marine ecosystems, predator population trends are thought to integrate the state of lower trophic levels and the physical environment that they inhabit [4, 5]. Consequently, predator population trends are often considered to be good indicators of ecosystem health [6].

The burgeoning human population, with disproportionately higher growth rates in coastal areas, exerts increased pressure on coastal ecosystems and marine species [7]. Coastally distributed dolphin species are particularly susceptible to current and future human-related threats such as habitat degradation, overfishing of prey species, and bycatch in fishing gear or shark exclusion nets [8, 9]. Examples of inshore dolphin species that are faced with multiple anthropogenic threats include the vaquita (*Phocoena sinus*) [10], humpback dolphins (*Sousa spp)* [11, 12], Australian snubfin dolphins (*Orcaella heinsohni)* [13], Hector’s dolphins (*Cephalorhynchus hectori*) [14] and the Indo-Pacific bottlenose dolphin (*Tursiops aduncus)* [15]. Studies that document population size and trends are essential for conservation and management of such species [16].

**Fig 1:**
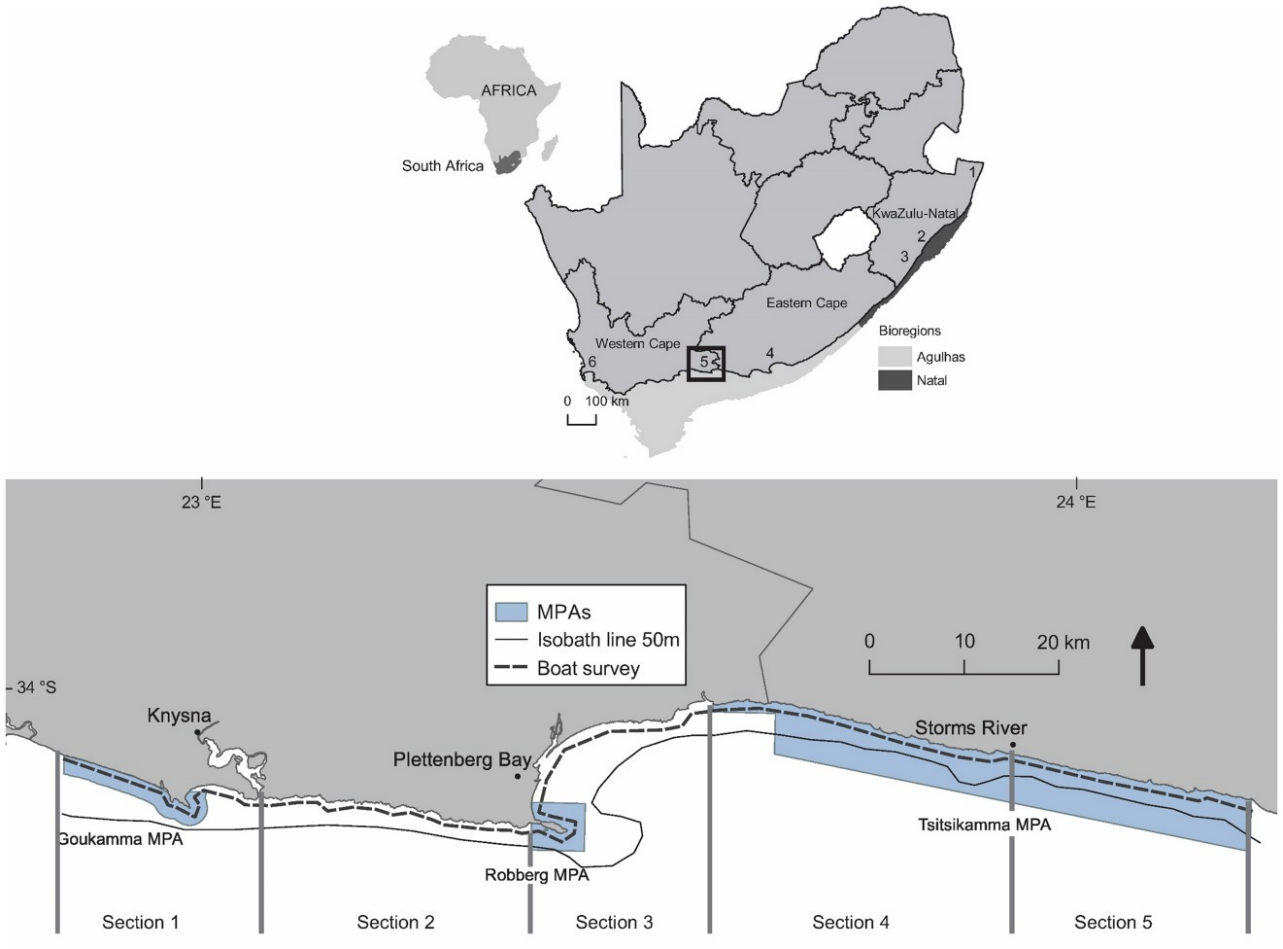
Map of South Africa and the study area, which extended from the western boundary of the Goukamma MPA to the eastern boundary of the Tsitsikamma MPA. Boat surveys were conducted approximately 100 m from the coast (dashed black line). Locations and bioregions mentioned in the text are shown on the South African map: (1) Kosi Bay; (2) Durban; (3) Ifafa (all within the Natal Bioregion). (4) Algoa Bay; (5) study area; (6) False Bay (all within the Agulhas Bioregion).

*T*. *aduncus* is listed as Near Threatened, globally, by the IUCN Red List of Threatened Species [15]. Their distribution is apparently continuous along coastal areas (including mid-ocean island shores) in the Indian Ocean, from False Bay (South Africa) eastwards to the Solomon Islands and New Caledonia in the western Pacific Ocean [17], including the east and west coasts of Australia and the south-east Asian waters [18]. The most recent Red List of Mammals of South Africa (published in 2016) [19] recognized three sub-populations of *T*. *aduncus* in South African waters [20]. A resident sub-population in northern KwaZulu-Natal (between Kosi Bay and Ifafa) was classified as Vulnerable; a migratory sub-population that is thought to move between Plettenberg Bay and Durban as Data Deficient; and a resident sub-population south of Ifafa with its western limit at False Bay as Near Threatened [19] (Fig 1). Research priorities identified for this species in South Africa and outlined in the Red List [19] include: 1) conducting research into their population genetics to establish significant management units; 2) assessing the effectiveness of Marine Protected Areas (MPAs) in addressing conservation needs of sub-populations; and 3) determining abundance estimates at local and regional (range-wide) scales [19]. Recent genetic studies [21, 22] identified two well-defined conservation units along the South African Coast: one along the Natal Bioregion and another in the Agulhas Bioregion (Fig 1). The conservation status of these newly defined conservation units is unknown.

Abundance trends of *T*. *aduncus* along South Africa’s coast is poorly understood. Estimates of population size are restricted to localised areas (summarized in [16]) and data on population trends are non-existent. For the sub-population in the Agulhas Bioregion, only two capture-recapture abundance estimates are available: one in Algoa Bay (1991–1994) where 28,482 (95% CI: 16,220–40,744) individuals were estimated [23] and another for Plettenberg Bay (2002–2003) where 6,997 (95% CI: 5,230–9,492) individuals were estimated [24]. These studies found that many individuals were utilising both bays, indicating a dynamic population on the south coast of South Africa with long-range movements [23].

To improve baseline population data and our understanding of the conservation status of *T*. *aduncus*, in South Africa, this study used photo-identification data collected over a two-year period to estimate *T*. *aduncus* population abundance along 145 km of coastline in the Agulhas Bioregion off the south coast of South Africa. We present group sizes, numbers of individuals photo-identified, and capture-recapture estimates of survival and abundance. We furthermore estimate these population parameters for a subset of the study area (Plettenberg Bay; 29 km of coastline) so that our results can directly be compared with a previous study restricted to this area that was conducted more than ten years earlier (2002–2003). This is the first attempt at assessing change in measures of abundance over time of a *T*. *aduncus* population within the Agulhas Bioregion.

## Methods

### Ethics statement

The project was permitted in terms of research permits RES 2013–67 and RES 2015–79, issued by the DEA, and animal ethics clearance A13-SCI-ZOO-001, issued by Nelson Mandela University.

### Study area, survey design and data collection

Data were collected during standardized boat surveys along 145 km of coastline within the Agulhas Bioregion, between the western border of the Goukamma MPA and the eastern boundary of the Tsitsikamma MPA on the south coast of South Africa (Fig 1). Ninety-seven kilometres of the study area coastline is within MPAs, namely the Goukamma, Robberg and Tsitsikamma MPAs. Boat-based whale and dolphin watching are not allowed in these MPAs. Also, all are closed to boat-based angling, but recreational shore angling is currently allowed in the Goukamma and Robberg MPAs, and in three controlled take-zones of the Tsitsikamma MPA, comprising about 20% of its shoreline [25–27]. The latter was a no take MPA during the study period.

There are two main dolphin space-use hotspots in the Goukamma MPA and the Plettenberg Bay area [28]; both characterized by sandy shores and gentle slopes. Coastal development within the study area is largely limited to the two half-heart bays, Buffels Bay (10 km from Knysna) and Plettenberg Bay. The exposed rocky coast with steep gradients that stretch between Knysna and Plettenberg Bay, and along the Tsitsikamma MPA, are largely uninhabited by humans.

The study area was surveyed from March 2014 to February 2016. Boat surveys followed a transect line running parallel to and approximately 100 m from the coastline (S1 Fig) thereby surveying the preferred habitat of *T*. *aduncus* [29–31]. A survey was defined as any research trip departing from a launch site at any given day (i.e., not as the traverse of a full transect). Regular surveys were conducted with the aim to traverse the entire study area transect two times every month (S1 Table). Due to the large extent of the study area, three different launch sites (Knysna, Plettenberg Bay and Storms River) were used. The study area was thus divided into five sections according to launch site and these sections were generally surveyed on different dates: Section 1 was from the western boundary of the Goukamma MPA to Knysna (length 24 km); Section 2 from Knysna to the western boundary of the Robberg MPA (34 km); Section 3 from the western boundary of the Robberg MPA to the western boundary of the Tsitsikamma MPA (29 km); Section 4 from the western boundary of the Tsitsikamma MPA to the Storms River mouth (31 km); and Section 5 from the Storms River mouth to the eastern boundary of the Tsitsikamma MPA (27 km) (Fig 1). Surveys in Sections 1, 2 and 3 were conducted using chartered vessels (7.9 m) equipped with two outboard engines (115 to 150 hp). For Sections 4 and 5, a rigid inflatable boat (5.5 m or 7.6 m) equipped with two outboard engines (70 hp or 100 hp) was used [28].

At least two experienced observers searched for dolphins during surveys which were performed at a constant speed of approximately eight knots. Survey effort was measured as the number of hours travelled in good sighting conditions (Beaufort scale ≤ 3). Survey effort was discontinued when conditions exceeded Beaufort scale 3 and also during encounters with dolphins or while the boat was in transit. While on transect, observers scanned for dolphins out to approximately 150 m from the boat. Once dolphins were located, they were approached at low speed so that the species, GPS location, group size, group composition and behaviour could be recorded, and to allow photographs of the dorsal fins of dolphins to be taken. After encounters, survey effort was usually resumed on the transect near where dolphins were initially encountered [28].

When dolphin groups were encountered, digital dorsal fin photo-ID images were taken using Nikon or Canon SLR cameras equipped with a Tamron 300 or 600 mm lens. Our sampling protocol aimed to photograph a random sample of individuals (in large groups) or all dolphins in the group (typically groups with less than 15 individuals). The dorsal fins of as many dolphins as possible were thus photographed from both sides (if possible), without any preference towards individuals with obvious markings [1]. Group sizes were estimated by the observers as minimum, maximum and best estimates, with best estimates not necessarily being the mean of the upper and lower estimates [32]. A group was defined as two or more animals within a 100 m radius of each other, showing similar behaviour [33].

### Data processing and analysis

#### Photo-identification catalogue and data selection

Dorsal fin images were cropped and graded according to the photograph quality (Q) and distinctiveness (D) by the lead author. Quality was scored from 1 to 3 (Q1 being excellent quality and Q3 poor quality). The Q grade was based upon photograph clarity, contrast, angle, portion of frame filled by the fin, angle, exposure, water spray and the percentage of the dorsal fin that is visible in the frame (adapted from [1, 34]). Photographs graded Q1 were therefore well exposed, without water droplets, in sharp focus, with the dorsal fin orientated perpendicular to the photographer and occupying a large proportion of the frame. Using only photographs graded Q1-Q2, the dorsal fins were then graded according to distinctiveness (D). Distinctiveness was graded from 1 to 3 (D1 being very distinctive and D3 without any distinctive characteristics). Photographs with distinctiveness grades D1-D2 were catalogued according to the location of the most prominent or distinguishing feature. The categories included: leading edge, mutilated, peduncle and trailing edge; with the latter subdivided into entire, low, mid or upper third. Dolphins were identified based on long lasting markings and as many features as possible [35]. Two experienced researchers visually compared photographs from each category to avoid misidentification of individuals (first within the same category and subsequently between categories where required).

#### New and repeat identifications and discovery curve

To evaluate whether the population had been sampled comprehensively, the cumulative number of newly identified individuals was plotted over time in a discovery curve. A discovery curve that reaches an asymptote indicates that the whole population has been identified and that it is likely to be a closed population [1]. The discovery curve of an open population (where births, deaths, immigration or emigration occurs) is not likely to reach an asymptote (e.g., [23]). Sighting frequencies of individual dolphins were calculated by dividing the number of individuals seen more than once by the total number of identified individuals. Sighting frequencies are therefore a measure of repeat observations of individuals that may, under adequate survey effort, provide some information on residence patterns (e.g., [36]).

#### Capture-recapture analysis

Only high quality photographs (Q ≤ 2) were used to construct encounter histories for all the identified individuals (D ≤ 2) using calendar month as capture occasion. Prior to capture-recapture analysis, the goodness-of-fit (GOF) of the fully time-dependent Cormack-Jolly-Seber (CJS) model was assessed in program U-CARE 2.2.2 [37] to verify whether the encounter histories met model assumptions [38]. This is an important first step in the analysis of capture-recapture data as the model selection approach we used for statistical inference assumed a general model that adequately fits the data. The underlying assumptions of the CJS model are that marks are long-lasting, individuals are not misidentified, sampling is instantaneous relative to the interval between occasions, and that survival and detection probabilities are homogeneous among marked animals that behave independently [38]. We tested the assumptions of homogeneous survival and detection probabilities using component tests in U-CARE. Test3.SR tests for transience (i.e., different future detection probabilities between newly identified and previously identified individuals), whereas Test2.CT tests for between-individual heterogeneity in detection (e.g., trap-happiness or shyness *sensu lato*) [38].

#### Estimating abundance in the study area

We fitted open population capture-recapture models (the POPAN parameterization of the Jolly-Seber model; [39]) using the software MARK 8.2 [40] to estimate the population size of *T*. *aduncus* in the study area. These models estimate the population size of marked individuals (${\hat{N}}_{m}$), apparent survival probability (ɸ), capture probability (p), and the probability of entry (b) from a “super-population” to the local population present in the study area. The relative support for each model we fitted to the data was obtained from Akaike Information Criterion (AICc) values [41]. Models with the lowest AICc values are the most parsimonious; model parsimony worsen gradually as ΔAICc (the difference between the model with the lowest AICc score and the model in question) increases. Models with a difference of more than 7 AICc units indicate strong support for the model with the lower AICc value [41]. Demographic parameters were designated as time dependent (t), constant over time (.) or seasonal (s), whereas capture probability were additionally allowed to vary with survey effort (hours surveyed per month). Seasons were defined as the austral winter (May-October) or summer (November-April) [42]. Test3.SR of the goodness-of-fit tests was significant (see Results), which represents a transient effect in encounter histories (many individuals are never detected in the months that follow the month that they were first captured). To account for this effect, we always used two-age classes when analysing survival probabilities (where the first age class represents apparent survival in the month following initial capture, and the second age class the subsequent apparent survival). Monthly survival probabilities estimated by the most parsimonious model were transformed to annual survival probability (ϕ_*annual*_ = ϕ_*month*_^12^) with associated variances re-scaled using the Delta method [43].

#### Plettenberg Bay comparison

Photo-identification data collected in Plettenberg Bay during 2002–2003 were previously analysed with closed population capture-recapture models (in the program CAPTURE [44, 45]) to estimate *T*. *aduncus* abundance [24]. The encounter histories (or photo-identification catalogue) from [24] were not available to us for re-analysis with more appropriate open population models. Consequently, we fitted similar models to the Plettenberg Bay subset of our data (section 3 of the transect) to facilitate the most direct comparison between the abundance estimates obtained from the two study periods. Though this comparison is impeded by the absence of the 2002–2003 data, and the modelling approach is subject to violations of population closure, we consider the comparison adequate for an approximation of relative (rather than absolute) change in abundance of *T*. *aduncus* in Plettenberg Bay. Note, therefore, that all closed population models (fitted to 2002–2003 and 2014–2016 data) overestimated Plettenberg Bay abundance, because lack of population closure would negatively bias capture probability estimates [44]. We estimated abundance from different closed population models fitted using program CAPTURE (for direct comparison to [24]), and also derived abundance for Plettenberg Bay using Huggins’ conditional likelihood models [46] in MARK. Abundance estimates may vary between CAPTURE and MARK as not all models are likelihood based in the former. In CAPTURE, model selection was based upon the model selection criteria values produced by the program [44], and not AICc. In this case, inference should only be based on models with selection criteria values ≥ 0.75 to 1 [44, 45]. The Huggins’ conditional likelihood models were ranked according to AICc, and always assumed the probability of first capture (p) to be the same as the probability of recapture (c) as no behavioural changes were expected following initial photographic capture [1].

#### Estimating total population size

The open and closed population capture-recapture abundance estimates refer only to the number (${\hat{N}}_{m}$) of distinctly marked individuals (D1 and D2) in the population. To estimate the total abundance of *T*. *aduncus*, ${\hat{N}}_{m}$ was adjusted to account for the proportion of unmarked individuals (D3) in the population [47]. The proportion of marked individuals (theta, $\hat{\theta }$) in the population was estimated as the ratio of distinctive individuals (D1 + D2) to the total sample (D1 + D2 + D3) present in good quality photographs (Q1 and Q2) [47]. Theta was calculated per month and then averaged over the study period. The total abundance (${\hat{N}}_{total}$) was estimated as:

$${\hat{N}}_{total}=\frac{{\hat{N}}_{m}}{\hat{\theta }}.$$

The standard error of the total population size was derived using a modification of the delta method [48]:

$$SE({\hat{N}}_{total})=\sqrt{{\hat{N}}_{total}^{\;2}(\frac{S{E({\hat{N}}_{m})}^{2}}{{\hat{N}}_{m}^{\;2}}+\frac{1-\hat{\theta }}{n\hat{\theta }})}$$

where ${SE(\hat{N}}_{m)}$ is the standard error of the marked population and *n* is the total number of animals from which $\hat{\theta }$ was estimated. Log-normal 95% confidence intervals for total population size were calculated as:

$$C\;=\;exp(1.96\sqrt{ln(1+\;{\left(\frac{{SE(\hat{N}}_{total})}{{\hat{N}}_{total}}\right)}^{2})})$$

with a lower confidence limit of ${\hat{N}}_{total}/C$ and an upper confidence limit of ${\hat{N}}_{total}\times C$ [49].

## Results

In total, 662.3 h of survey effort was conducted over 189 surveys and 145 days from March 2014 to February 2016 (S1 Table, S1 Fig). Individuals were encountered throughout the year but group sizes tended to vary by season. Average group size was estimated as 47 ± 55 (mean ± standard deviation (SD)) individuals, with larger group sizes during winter (57 ± 63) compared to summer (35 ± 42) (Mann-Whitney U test: U = 2694.5, p = 0.004; Table 1). For Plettenberg Bay only, we estimated a mean group size of 26 ± 26 individuals that did not vary by season (U = 301, p = 0.251; Table 1). Smaller groups thus occurred in Plettenberg Bay during 2014–2016 than elsewhere in the study area (U = 3677.5, p = 0.006). Our 2014–2016 Plettenberg Bay group size estimate was approximately 78% lower than that reported for 2002–2003 (Table 1).

**Table 1:** *T*. *aduncus* group size statistics for the entire research area (between the Goukamma and the Tsitsikamma marine protected areas), and Plettenberg Bay. Past estimates for Plettenberg Bay (2002–2003) are also given.

	Summer	Winter	Overall
Entire study area 2014–2016			
# surveys	99	90	189
search effort	342.7 h	319.6 h	662.3 h
# encounters	82	88	170
Mean ± SD	35 ± 42	57 ± 63	47 ± 55
Range	1–300	1–350	1–350
Median	20	40	30
Plettenberg Bay 2014–2016			
# surveys	30	25	55
search effort	116.6	101.6	218.2
# encounters	37	20	57
Mean ± SD	26 ± 28	26 ± 18	26 ± 26
Range	1–100	3–65	1–100
Median	15	23	18
Plettenberg Bay 2002–2003 [24]			
Mean ± SD	124 ± 111 ^1^	82 ± 143 ^1^	120 ± *NA* ^3^
	211 ± 139 ^2^	56 ± 76 ^2^	
Range	*NA*	*NA*	2–500 ^3^
Median	*NA*	*NA*	80 ^3^

*‘NA’*: not available;

^1^ in 2002;

^2^ in 2003;

^3^ in 2002–2003.

We spent 80.6 h with *T*. *aduncus* groups during surveys, with a mean of 0.47 h per encounter (± 0.48 h, range: 0–2.45 h). A total of 10,431 dorsal fin photographs were taken during encounters, of which 1,569 (15%) were of acceptable quality grade (≤ Q2). The ≤ Q2 subset contained 1,323 (12.7%) photographs with distinctive individuals (≤D2) and the final catalogue consisted of 817 identified animals. The proportion of distinctively marked individuals (adults and juveniles) averaged over all months was 0.82 (±0.11) for the entire study area and 0.82 (±0.23) for Plettenberg Bay.

**Fig 2:**
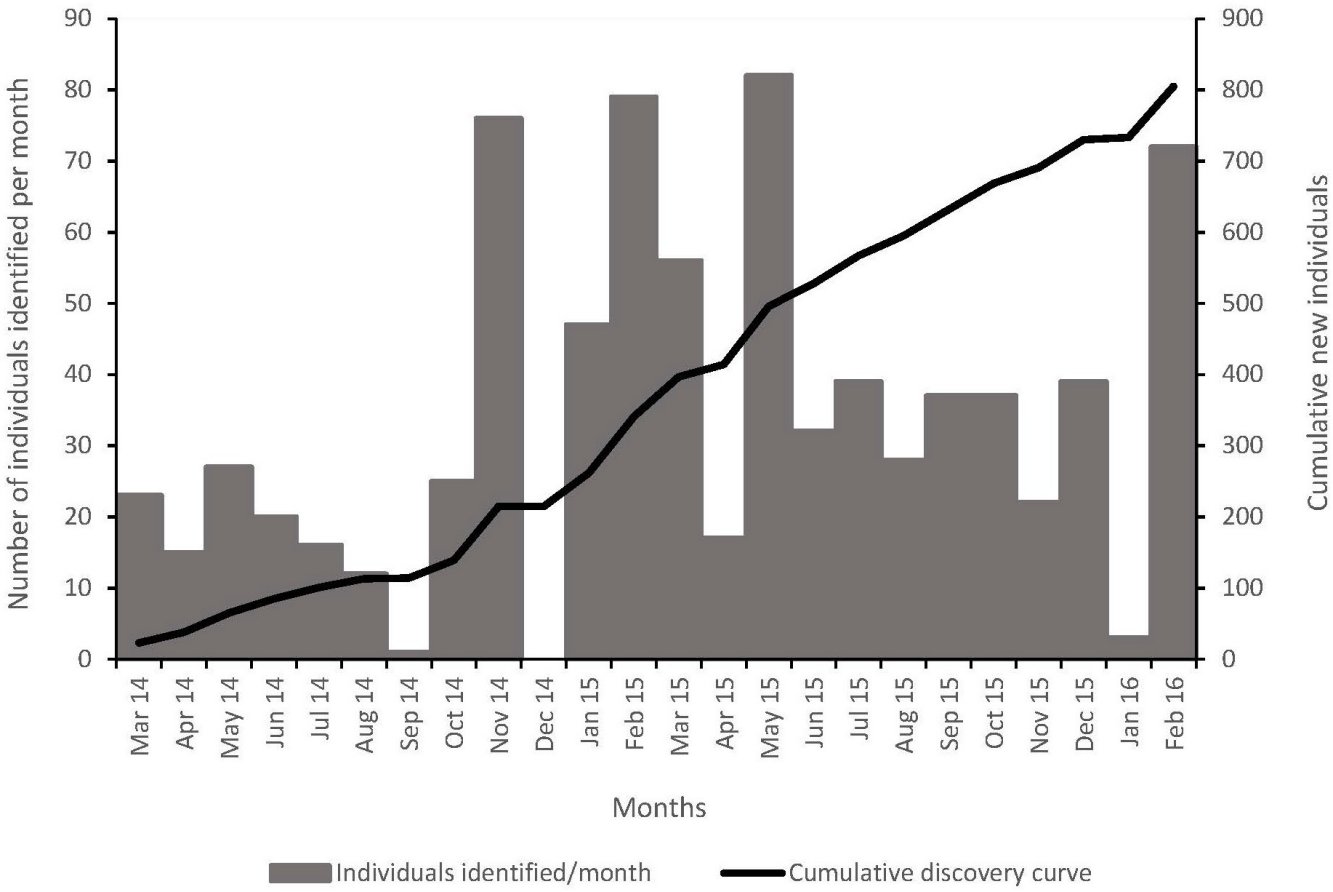
The number of new *T*. *aduncus* individuals identified from photographs per month (bar plot), and the cumulative discovery curve (per survey) for new individuals (black line). In total, 189 surveys were conducted from March 2014 to February 2016.

The discovery curve never reached an asymptote (Fig 2). New individuals were thus still being identified towards the end of the study period, indicating either that the population is open or that not all individuals of a closed population had been identified. Of the identified animals, a high proportion (72.7%) of individuals were encountered only once. The sighting frequency of those individuals seen more than once (27.3% of the individuals) was 16.8% encountered twice; 6.2% encountered three times; and 4.3% encountered between 4 and 7 times.

### Population size in the study area

Test3.SR of the goodness-of-fit test (Table 2) indicated a transient effect in the encounter history data. By including age structure in the survival parameter, we could recalculate the goodness-of-fit statistic without the contribution of Test3.SR. The resultant test (‘Transient model’, Table 2) suggested that the model fit was adequate, as Test2.CT did not detect capture heterogeneity. The most parsimonious POPAN model for the entire study area assumed constant survival (with two age classes), time dependent capture probability, and a seasonal (summer and winter) probability to enter the local population from the “super-population” (Table 3 and S2 Table for the full table). This model predicted a population size estimate (${\hat{N}}_{m}$) of 1,765 (95% CI: 1,535–2,029) marked individuals, which translates to a total population of 2,155 (95% CI: 1,873–2,479) individuals. All other models with AICc support produced similar population size estimates. Apparent annual survival was estimated to be 0.87 (95% CI: 0.53–0.97) while mean probability of entry for this model was 0.04 (0.03–0.07) in summer and zero (parameter on the boundary) in winter.

**Table 2:** Goodness-of-fit results for the Cormack-Jolly-Seber model fitted to individual sighting histories of *T*. *aduncus* (2014–2016).

Test	*χ*^2^	df	p	$\hat{c}$
Test3.SR	**61.83**	**19**	**<0.001**	
Test3.SM	9.51	18	0.947	
Test2.CT	17.59	17	0.415	
Test2.CL	28.11	22	0.172	
CJS Model	**117.04**	**76**	**0.002**	1.54
Transient model	55.20	57.00	0.543	0.97

Parameters are: Chi-squared statistic (*χ*^2^), degrees of freedom (df), statistical significance (p-value) and variance inflation factor (ĉ).

Significant *χ*^2^ statistics (p < 0.05) are in boldface.

**Table 3:** Model selection and abundance estimates for *T*. *aduncus* obtained from POPAN open population Jolly-Seber models.

Model^1^	NP	Model selection criteria				Marked population				Total population			
		AICc	ΔAICc	W	Dev	${\hat{N}}_{m}$	SE	LCL	UCL	${\hat{N}}_{total}$	SE	LCL	UCL
**ɸ(a)p(t)b(s)N(.)**	**29**	**2811.4**	**0.0**	**0.4**	**-3722.5**	**1765**	**126**	**1535**	**2029**	**2155**	**154**	**1873**	**2479**
**ɸ(a+s)p(t)b(s)N(.)**	**30**	**2812.2**	**0.8**	**0.3**	**-3723.9**	**1764**	**109**	**1562**	**1992**	**2154**	**134**	**1906**	**2434**
**ɸ(a)p(t)b(.)N(.)**	**28**	**2813.3**	**1.9**	**0.2**	**-3718.6**	**1761**	**140**	**1507**	**2058**	**2150**	**172**	**1839**	**2514**
**ɸ(a+s)p(t)b(.)N(.)**	**29**	**2814.4**	**3.0**	**0.1**	**-3719.5**	**1772**	**127**	**1540**	**2038**	**2163**	**155**	**1879**	**2489**
ɸ(a+t)p(t)b(s)N(.)	51	2850.1	38.7	0.0	-3731.1	1796	108	1597	2020	2193	132	1948	2468

Models are ordered according to their Akaike Information Criterion (AICc) values (only the top five models are shown; the full model set is available online (S2 Table)). Column headings are: number of parameters (NP); AICc; the difference between the current model and the top ranked model (ΔAICc); the relative AICc support for the model (W); the model deviance (Dev); estimate of the number of marked animals (${\hat{N}}_{m}$); standard error of ${\hat{N}}_{m}$ (SE); lower and upper limits of the 95% confidence interval of ${\hat{N}}_{m}$ (LCL and UCL); and estimated total population size (${\hat{N}}_{total}$).

^1^ The parameters used to build these models are: survival probability (ɸ); capture probability (p); entry probability (b); population size (N). Each parameter may be designated as age class dependent (a), time dependent (t), constant over time (.) and seasonal (s).

### Plettenberg Bay comparison

Capture probability varied by month according to program CAPTURE’s closed population models for Plettenberg Bay (2014–2016). Model M(t) predicted a total population size (${\hat{N}}_{total}$) of 1,292 (95% CI: 1026–1,626) individuals (Tables 4 and 5). Model M(th) (which assumed heterogeneous capture probabilities in addition to time dependence, and had a model selection criteria value above 0.75) predicted a total abundance of 1,815 (95% CI: 1,355–2,432) individuals (Tables 4 and 5). Phillips [24] derived a total population estimate of 6,997 for Plettenberg Bay in 2002–2003 [24] using an M(th) model structure. This corresponds to a more than three times larger total population estimate in 2002–2003 than in 2014–2016.

**Table 4:** Comparison of the model selection criteria values produced by program CAPTURE in MARK for the study period (2014–2016) and 2002–2003 [24].

Criteria/Model^1^	M(0)	M(t)	M(b)	M(h)	M(bh)	M(th)	M(tb)	M(tbh)
Plettenberg Bay (2014–2016)	0.13	1	0.13	0	0.07	0.76	0.34	0.24
Plettenberg Bay (2002–2003)^2^	0.18	0.61	0	0.05	0.21	1	0.42	0.53

The higher the selection criteria value, the better the model fits the data, with a maximum value of one [44].

^1^ M(0) = constant probability of capture (p); M(t) = time-varying p; M(b) = p influenced by behavioural response to capture; M(h) = among-individual heterogeneity in p. Models M(bh), M(th), M(tb) and M(tbh) combine different effects [50].

^2^ Results extracted from [24].

**Table 5:** Estimates of *T*. *aduncus* abundance in Plettenberg Bay derived from closed population models in program CAPTURE for the study period (2014–2016) and 2002–2003 [24].

Model ^1^	Marked population			Total population		
	${\hat{N}}_{m}$	LCL	UCL	${\hat{N}}_{total}$	LCL	UCL
Plettenberg Bay (2014–2016)						
M(t)	1063	858	1360	1292	1026	1626
M(th)	1494	1131	2024	1815	1355	2432
Plettenberg Bay (2002–2003) ^2^						
M(th)	4833	3612	6556	6997	5230	9492

Column heading are: marked population size (${\hat{N}}_{m}$) and total population size (${\hat{N}}_{m}$) estimates; lower and upper limits of 95% confidence intervals (LCL and UCL).

^1^ M(t) = time-varying capture probability (p); M(h) = among-individual heterogeneity in p; M(th) = a combination of the above.

^2^ Results extracted from [24].

The most parsimonious Huggins’ conditional likelihood closed population model for Plettenberg Bay (2014–2016) assumed equal, but time dependent, capture and recapture probabilities. The abundance estimate derived from this model ${\hat{N}}_{total}=1,297$ (95% CI: 1,030–1,632 individuals; Table 6) was similar to that estimated using program CAPTURE.

**Table 6:** Estimates of *T*. *aduncus* abundance in Plettenberg Bay derived from Huggins’ conditional likelihood models for the study period (2014–2016).

Model^1^	Model selection criteria					Marked population			Total population		
	NP	AICc	ΔAICc	Dev	W	${\hat{N}}_{m}$	LCL	UCL	${\hat{N}}_{total}$	LCL	UCL
*Plettenberg Bay (2014–2016)*											
p = c(t)	24	1983	0	3400	1	1067	859	1357	1297	1030	1632
Pi p = c(t)	49	2034	50	3400	0	1067	859	1357	1297	1030	1632
p = c(.)	1	2709	726	4172	0	1227	979	1571	1491	1175	1892
Pi p = c(.)	3	2713	730	4172	0	1227	979	1571	1491	1175	1892

Column heading are the same as in Table 3.

^1^ p = initial capture probability c = recapture probability Pi = heterogeneous p and c; t = time, (.) = constant.

## Discussion

The current poor understanding of *T*. *aduncus* abundance and population trends in South African waters, and large parts of this coastal dolphin’s global range, hampers conservation assessments of this species [15, 19]. Though more data are needed to confirm *T*. *aduncus* population trends in South Africa, this study contributes new abundance information that can assist conservation management. We identified 817 individuals during two years of field surveys, and open population capture-recapture estimates yielded a total population size of 2,155 (95% CI: 1,873–2,479) individuals. Even the minimum number of identified individuals is relatively large compared to many other coastal populations of *T*. *aduncus* in the Indo-Pacific region, which often number in the low hundreds of individuals or fewer [15, 51]. Sighting frequencies indicated that only 27% of individuals were identified in more than one month. This transience effect suggests that individuals observed within the study area are part of an open population that ranges more widely, at least as far as Algoa Bay [23] (approximately 200 km to the east). We recorded smaller group sizes and an apparent decline in abundance in the Plettenberg Bay subset of the study area compared to estimates obtained in 2002–2003 using similar methods [24]. While the drivers of the apparent decline are unknown, our results highlight the need for long-term monitoring efforts to improve conservation assessments.

Knowledge of abundance and distribution are essential for understanding the population dynamics of animal species. However, estimating population size and temporal trends in abundance for cetaceans can be particularly challenging [52]. We used photo-identification of natural markings and capture-recapture analysis to estimate the population size of *T*. *aduncus* in the Agulhas Bioregion of South Africa. Our total population estimate (2,155; 1,873–2,479) for the study area between the Goukamma and Tsitsikamma MPAs on the south coast of South Africa is roughly thirteen times smaller than that derived for Algoa Bay (28,482; 16,220–40,744) from data collected in the early 1990s [23]. Although the current size of the Algoa Bay *T*. *aduncus* population could be very different, large group sizes (ranging from 200–600 individuals) still occur here [53]. We observed smaller group sizes, and our entire study area population size estimate is approximately a third of that obtained during a previous study [24] that focused only on Plettenberg Bay (6,997; 5,230–9,492 in 2002–2003) based on 637 individually identified animals.

Lack of data generally prohibits assessment of *T*. *aduncus* population trends along the South African coast, but the existence of past estimates for Plettenberg Bay allowed us to compare changes in both group size and abundance for this section of our study area, between 2002–2003 [24] and 2014–2016. A comparison of closed population size estimates between the two periods suggests a decrease in abundance of well over 70% in Plettenberg Bay. We caution that these numbers are approximate measures or proxies of true population size, which should be interpreted cognisant of their limitations. These include slight differences in field methodology (discussed later), and model shortcomings such as violations of population closure. Lack of population closure negatively biases capture probability estimates, thereby causing abundance to be overestimated [44]. Thus, the closed model abundance estimates we report for Plettenberg Bay are undoubtedly biased high because of violation of population closure. We assume that both the 2002–2003 and 2014–2016 estimates are similarly biased, and thus can be compared to each other to provide a measure of relative change. That said, dolphin group sizes (which can be a sign of changes in abundance [54]) also decreased strongly (by nearly 80%, from around 120 on average in 2002–2003 [24] to approximately 26 in 2014–2016) in Plettenberg Bay. The mean group size estimated for Plettenberg Bay in this study was considerably lower than that recorded for the entire study area (47 ± 55). Nonetheless, even the latter estimate is 60% lower than group sizes observed in 2002–2003. The declining group size trend is further supported by a shore-based group size estimate of 140 individuals from the early 1970s [55]. It is worth noting, however, that our group sizes for 2014–2016 are comparable to those elsewhere in the species range (generally less than 30 individuals [56]) but smaller than past estimated and the mean group size commonly observed in Algoa Bay [23, 53].

### Hypotheses for Plettenberg Bay decline

Understanding the causes for the apparent changes in dolphin abundance and group sizes in the Plettenberg Bay subset of the study area will aid conservation management strategies. Although the causes of apparent decline are unknown, we discuss anthropogenic pressure, ecosystem change and methodological inconsistencies as plausible hypotheses. Like other coastally distributed dolphin species worldwide, *T*, *aduncus* populations are vulnerable to a variety of anthropogenic pressures. The Bitou municipality (which includes Plettenberg Bay) is the fastest growing municipality in South Africa’s Western Cape Province, with an average annual population growth of 4.8% from 2001 to 2013 (Western Cape Government 2014). We may therefore expect anthropogenic pressures such as coastal development and pollutants [57] to have increased within the study area during this time. Vessel traffic (including tourism activities, such as boat-based marine mammal viewing and fishing charters) [58–60] is another potential anthropogenic pressure that may have increased along with a burgeoning tourism industry. The impacts of tourism on animal populations is generally measured by short-term behavioural responses (e.g., [59]), yet evidence is mounting that disturbance caused by these activities can have long-term demographic implications in some species [54]. Close approaches by boats, for example, may impede feeding and resting behaviour [61] or sufficiently disturb dolphins to induce shifts in residency patterns or regional abundance [26]. However, not all dolphin populations respond equally to vessel disturbance, and small, closed populations that are unable to avoid regular disturbance are typically more sensitive than open populations like those that occur within our study area [62]. The open population character of the *T*. *aduncus* population along the Agulhas Bioregion of South Africa may thus reduce its sensitivity to certain anthropogenic pressures.

Declines in marine predator populations or shifts in group size, habitat use and distributional range, can potentially be indicative of ecosystem changes [63]. Changes in *T*. *aduncus* prey resources may have occurred in the Agulhas Bioregion in recent decades. One notable change in prey availability is the decline in the productivity of chokka squid *(Loligo vulgaris reynaudii)* [64, 65], a favoured prey species for *T*. *aduncus*, which spawns through most of the study area [66]. In addition, the prominence of South Africa’s sardine run, which is characterized by large schools of sardine (*Sardinops sagax*) moving northwards along the east coast during winter months, followed by vast numbers of predators including *T*. *aduncus* [67], has also declined since the early 2000s [68]. This could have resulted in a reduction in the number and potentially the size of transient dolphin groups traversing the study area in 2014–2016 to join the sardine run during the autumn-winter period.

Another important change in the Plettenberg Bay marine ecosystem since the early 2000s is the recolonization and rapid growth of the Cape fur seal (*Arctocephalus pusillus pusillus*) colony on Robberg Peninsula, which forms the southern boundary of the bay [69]. Increased fur seal abundance may firstly increase inter-specific competition for prey resources [64, 70]. Secondly, fur seal presence on the south coast of South Africa is associated with an increase in aggregations of white sharks (*Carcharodon carcharias)*, especially during winter [71, 72]. White sharks may impact the *T*. *aduncus* population though predation [73] or through perceived predation risk, i.e., by establishing a landscape of fear that modifies dolphin behaviour patterns. The influx of both sharks and seals could therefore conceivably have brought about changes in *T*. *aduncus* behaviour, manifested through changes in residency patterns or group sizes [74].

It is also necessary to consider differences in data collection between the two periods that could potentially have influenced the comparisons we made. The 2002–2003 study differed from the current one in that 1) surveys extended further offshore and not only followed the coast (because the surveys targeted all cetaceans and not only coastal dolphin species); 2) other vessels would communicate sightings to the research boat when dolphins were encountered; 3) film cameras were used for photo-identification. The first of these could bias towards lower detection probabilities of *T*. *aduncus* in the previous study, because of low encounter rates further offshore. On the other hand being alerted to groups by other vessels in the vicinity could favour higher detection probabilities. Notwithstanding the obvious advantages of digital versus film cameras, data collection and photo-identification procedures were similar between the two studies, although observer-specific partialities in the estimation of group sizes cannot be discounted. However, even the maximum group size estimates (*cf*. best group size estimate) in this study was smaller than the mean group size reported for 2002–2003. The differences that exist between the studies and observers are unlikely to account for the more than 70% reduction in estimated abundance and group size between the two periods.

### Research recommendations

We do not know whether the apparent decrease in measures of abundance in Plettenberg Bay between 2002–2003 and 2014–2016 represent local or regional declines, or distributional shifts to outside of the study area. The results of this study thus highlight the importance of assessing trends in abundance across the South African distributional range. While the causes of the apparent changes are not yet known, a multifaceted precautionary approach to prevent and mitigate threats to the population and also that of the sympatric and endangered Indian Ocean humpback dolphins *Sousa plumbea* is advised. As is the case for *T*. *aduncus*, a substantial decline in abundance and group size within our study area has also been reported for *S*. *plumbea*, which are known to be sensitive to anthropogenic threats [75–78]. Although the concurrent change in measures of abundance for both dolphin species suggest real declines, more data are needed to confirm population trends, and to determine the drivers of population change. Data that will allow causative mechanisms to be identified can only be obtained through long-term monitoring. Increased search effort will allow increased confidence in demographic parameter estimates, and could allow *T*. *aduncus* temporary emigration to be quantified (e.g., through robust design sampling and analysis). Satellite telemetry studies will augment these efforts to better understand residency, connectivity and fine scale movement of the *T*. *aduncus* populations in South Africa. Collaboration with local whale watching companies and non-profit organizations (NPO’s) can provide additional resources (e.g., platforms of opportunity) to help facilitate long-term monitoring of *T*. *aduncus* and other marine mammal populations. Such efforts are important, given that our data indicate that a relatively large population of *T*. *aduncus* (compared to many other coastal populations in the Indo-Pacific region [15, 51] occur in the study region.

Many of South Africa’s existing MPAs are in coastal waters, thus overlapping with the distribution of coastal dolphins such as *T*. *aduncus* [79]. Expanding current MPAs or establishing new ones has been recommended to assist population recovery of *S*. *plumbea* in South Africa [78]. Because of similarities in distribution, habitat preferences and threats between the two species, such measures would likely also benefit *T*. *aduncus* populations. However, due to the highly mobile nature of marine mammals, they tend to only temporarily occupy protected areas, and as such, MPAs on their own are not likely to be sufficient for their conservation, although they may reduce boat disturbance within their boundaries and help conserve prey species. Further research on potential anthropogenic or environmental factors that could have contributed to the observed changes in abundance and distribution is needed. Until such time that population trends and potential causative mechanisms behind population changes are better understood, a precautionary approach with regard to conservation management and potential impacts of anthropogenic pressures is recommended.

## Supporting information

S1 Fig Survey tracks along the study area. (TIF)

S1 Table Search effort per section of the study area, year and season. (DOCX)

S2 Table Model selection and abundance estimates for *T*. *aduncus* obtained from POPAN open population Jolly-Seber models. (DOCX)
