# Supplementary figures and images for: Abundance of Indo-Pacific bottlenose dolphins (Tursiops aduncus) along the south coast of South Africa
Source: PLoS One. 2020 Oct 12;15(10):e0227085. doi: 10.1371/journal.pone.0227085 (PMC7549814; doi:10.1371/journal.pone.0227085)

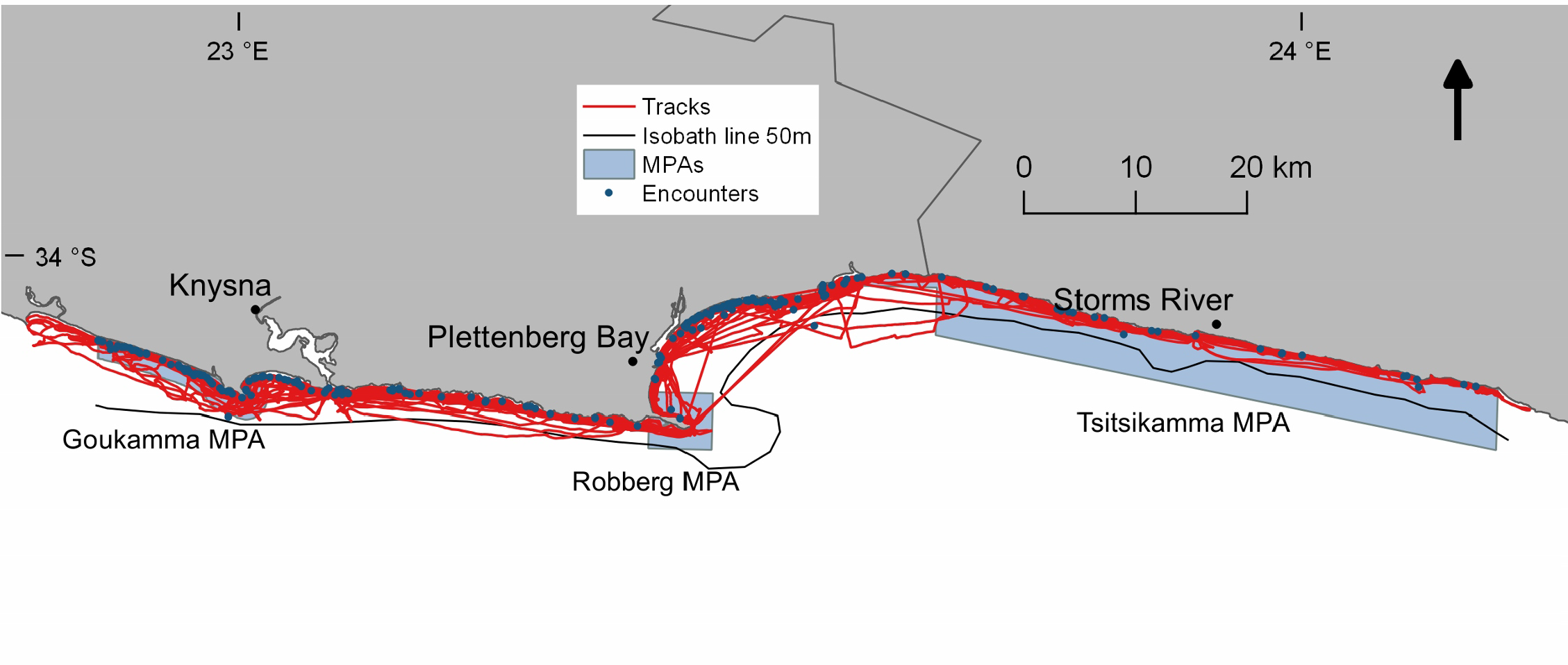

Supplement: S1 Fig — (TIF) [file pone.0227085.s001.tif]
