# Supplementary material for: Abundance of Indo-Pacific bottlenose dolphins (Tursiops aduncus) along the south coast of South Africa
Source: PLoS One. 2020 Oct 12;15(10):e0227085. doi: 10.1371/journal.pone.0227085 (PMC7549814; doi:10.1371/journal.pone.0227085)
Supplement: S1 Table — (DOCX) [file pone.0227085.s002.docx]

**S1 Table: Search effort per section of the study area, year and season.**

|  | Search effort (hours: minutes) | | | Number of surveys | | |
| --- | --- | --- | --- | --- | --- | --- |
| Year/Section* | Summer | Winter | Total | Summer | Winter | Total |
| 2014 | 142:33 | 139:08 | 281:41 | 42 | 42 | 84 |
| 1 | 33:17 | 25:13 | 58:30 | 10 | 10 | 20 |
| 2 | 30:49 | 31:32 | 62:21 | 9 | 10 | 19 |
| 3 | 53:07 | 43:54 | 97:01 | 14 | 12 | 26 |
| 4 | 17:58 | 19:57 | 37:55 | 6 | 5 | 11 |
| 5 | 7:22 | 18:32 | 25:54 | 3 | 5 | 8 |
| 2015 | 167:49 | 180:29 | 348:18 | 47 | 48 | 95 |
| 1 | 40:12 | 42:22 | 82:34 | 12 | 13 | 25 |
| 2 | 34:13 | 39:44 | 73:57 | 12 | 12 | 24 |
| 3 | 53:13 | 57:39 | 110:52 | 13 | 13 | 26 |
| 4 | 25:49 | 25:13 | 51:02 | 6 | 6 | 12 |
| 5 | 14:22 | 15:31 | 29:53 | 4 | 4 | 8 |
| 2016 | 32:19 |  | 32:19 | 10 |  | 10 |
| 1 | 9:17 |  | 9:17 | 3 |  | 3 |
| 2 | 8:45 |  | 8:45 | 3 |  | 3 |
| 3 | 10:15 |  | 10:15 | 3 |  | 3 |
| 4 | 4:02 |  | 4:02 | 1 |  | 1 |
| Total | 342:41 | 319:37 | 662:18 | 99 | 90 | 189 |

*Section 1: from the western boundary of the Goukamma MPA to the Knysna (length 24 km); Section 2 from the Knysna to the western boundary of the Robberg MPA (34 km); Section 3 from the western boundary of the Robberg MPA to the western boundary of the Tsitsikamma MPA (29 km); Section 4 from the western boundary of the Tsitsikamma MPA to the Storms River mouth (31 km); and Section 5 from the Storms River mouth to the eastern boundary of the Tsitsikamma MPA (27 km)
