# Supplementary material for: Abundance of Indo-Pacific bottlenose dolphins (Tursiops aduncus) along the south coast of South Africa
Source: PLoS One. 2020 Oct 12;15(10):e0227085. doi: 10.1371/journal.pone.0227085 (PMC7549814; doi:10.1371/journal.pone.0227085)
Supplement: S2 Table — (DOCX) [file pone.0227085.s003.docx]

**S2 Table: Model selection and abundance estimates for *T. aduncus* obtained from POPAN open population Jolly-Seber models.** Models are ordered according to their Akaike Information Criterion (AICc) values. Column headings are: number of parameters (NP); AICc; the difference between the current model and the top ranked model (ΔAICc); the model deviance (Dev); the relative support for the model (W); estimate of the number of marked animals ($\hat{N}_{m}$); lower and upper limits of the 95% confidence interval of (LCL and UCL); and estimated total population ($\hat{N}_{total}$).

|  |  | Model selection criteria | | | |  | Marked population | | | |  | Total population | | | |
| --- | --- | --- | --- | --- | --- | --- | --- | --- | --- | --- | --- | --- | --- | --- | --- |
| Model^1^ | NP | AICc | ΔAICc | W | Dev |  | $\hat{N}_{m}$ | SE | LCL | UCL |  | $\hat{N}_{total}$ | SE | LCL | UCL |
| **ɸ(a)p(t)b(s)N(.)** | **29** | **2811.4** | **0.0** | **0.4** | **-3722.5** |  | **1765** | **126** | **1535** | **2029** |  | **2155** | **154** | **1873** | **2479** |
| **ɸ(a+s)p(t)b(s)N(.)** | **30** | **2812.2** | **0.8** | **0.3** | **-3723.9** |  | **1764** | **109** | **1562** | **1992** |  | **2154** | **134** | **1906** | **2434** |
| **ɸ(a)p(t)b(.)N(.)** | **28** | **2813.3** | **1.9** | **0.2** | **-3718.6** |  | **1761** | **140** | **1507** | **2058** |  | **2150** | **172** | **1839** | **2514** |
| **ɸ(a+s)p(t)b(.)N(.)** | **29** | **2814.4** | **3.0** | **0.1** | **-3719.5** |  | **1772** | **127** | **1540** | **2038** |  | **2163** | **155** | **1879** | **2489** |
| ɸ(a+t)p(t)b(s)N(.) | 51 | 2850.1 | 38.7 | 0.0 | -3731.1 |  | 1796 | 108 | 1597 | 2020 |  | 2193 | 132 | 1948 | 2468 |
| ɸ(a+t)p(t)b(.)N(.) | 50 | 2852.7 | 41.3 | 0.0 | -3726.4 |  | 1800 | 115 | 1588 | 2040 |  | 2198 | 141 | 1938 | 2492 |
| ɸ(a+s)p(t)b(t)N(.) | 51 | 2853.0 | 41.6 | 0.0 | -3728.2 |  | 1738 | 124 | 1511 | 1998 |  | 2122 | 152 | 1844 | 2442 |
| ɸ(a)p(t)b(t)N(.) | 50 | 2854.2 | 42.8 | 0.0 | -3724.9 |  | 1722 | 126 | 1492 | 1987 |  | 2102 | 155 | 1820 | 2428 |
| ɸ(a+t)p(t)b(t)N(.) | 72 | 2900.3 | 88.8 | 0.0 | -3727.9 |  | 1742 | 113 | 1535 | 1977 |  | 2127 | 138 | 1873 | 2416 |
| ɸ(a+s)p(.)b(t)N(.) | 28 | 3246.6 | 435.1 | 0.0 | -3285.3 |  | 1866 | 107 | 1667 | 2088 |  | 2278 | 132 | 2034 | 2551 |
| ɸ(a+s)p(s)b(t)N(.) | 29 | 3248.6 | 437.2 | 0.0 | -3285.4 |  | 1869 | 109 | 1668 | 2094 |  | 2282 | 134 | 2035 | 2559 |
| ɸ(a+s)p(e)b(t)N(.) | 29 | 3252.5 | 441.1 | 0.0 | -3281.4 |  | 1924 | 133 | 1681 | 2203 |  | 2350 | 163 | 2051 | 2692 |
| ɸ(a)p(e)b(t)N(.) | 28 | 3256.5 | 445.1 | 0.0 | -3275.3 |  | 1922 | 140 | 1667 | 2216 |  | 2346 | 171 | 2034 | 2707 |
| ɸ(a)p(.)b(t)N(.) | 27 | 3257.3 | 445.9 | 0.0 | -3272.5 |  | 1851 | 119 | 1632 | 2098 |  | 2260 | 146 | 1992 | 2564 |
| ɸ(a)p(s)b(t)N(.) | 28 | 3259.4 | 447.9 | 0.0 | -3272.5 |  | 1848 | 120 | 1627 | 2098 |  | 2256 | 147 | 1986 | 2563 |
| ɸ(a+t)p(e)b(t)N(.) | 50 | 3275.5 | 464.1 | 0.0 | -3303.5 |  | 2009 | 118 | 1791 | 2254 |  | 2453 | 145 | 2185 | 2754 |
| ɸ(a+t)p(.)b(t)N(.) | 49 | 3276.7 | 465.3 | 0.0 | -3300.1 |  | 1966 | 112 | 1759 | 2197 |  | 2400 | 137 | 2146 | 2685 |
| ɸ(a+t)p(s)b(t)N(.) | 50 | 3278.7 | 467.3 | 0.0 | -3300.4 |  | 1961 | 111 | 1754 | 2192 |  | 2394 | 137 | 2140 | 2678 |
| ɸ(a+s)p(e)b(s)N(.) | 8 | 3282.8 | 471.4 | 0.0 | -3207.7 |  | 2143 | 127 | 1908 | 2407 |  | 2617 | 156 | 2328 | 2942 |
| ɸ(a+s)p(.)b(s)N(.) | 7 | 3285.2 | 473.8 | 0.0 | -3203.3 |  | 2138 | 110 | 1934 | 2364 |  | 2610 | 135 | 2359 | 2888 |
| ɸ(a+s)p(s)b(s)N(.) | 8 | 3285.3 | 473.9 | 0.0 | -3205.2 |  | 2140 | 110 | 1935 | 2366 |  | 2612 | 135 | 2361 | 2891 |
| ɸ(a)p(e)b(s)N(.) | 7 | 3286.0 | 474.5 | 0.0 | -3202.6 |  | 2121 | 118 | 1902 | 2365 |  | 2590 | 145 | 2321 | 2890 |
| ɸ(a)p(.)b(s)N(.) | 6 | 3287.1 | 475.7 | 0.0 | -3199.4 |  | 2130 | 118 | 1910 | 2375 |  | 2601 | 146 | 2331 | 2903 |
| ɸ(a)p(s)b(s)N(.) | 7 | 3289.0 | 477.6 | 0.0 | -3199.5 |  | 2125 | 119 | 1904 | 2372 |  | 2595 | 147 | 2323 | 2898 |
| ɸ(a+s)p(e)b(.)N(.) | 7 | 3296.1 | 484.6 | 0.0 | -3192.5 |  | 2111 | 127 | 1877 | 2375 |  | 2578 | 156 | 2290 | 2902 |
| ɸ(a+s)p(s)b(.)N(.) | 7 | 3299.3 | 487.9 | 0.0 | -3189.2 |  | 2097 | 113 | 1888 | 2330 |  | 2561 | 139 | 2303 | 2847 |
| ɸ(a+t)p(e)b(s)N(.) | 29 | 3300.4 | 489.0 | 0.0 | -3233.6 |  | 2228 | 132 | 1983 | 2503 |  | 2720 | 163 | 2419 | 3058 |
| ɸ(a+s)p(.)b(.)N(.) | 6 | 3302.0 | 490.6 | 0.0 | -3184.5 |  | 2088 | 112 | 1880 | 2319 |  | 2549 | 138 | 2293 | 2834 |
| ɸ(a+t)p(.)b(s)N(.) | 28 | 3302.1 | 490.7 | 0.0 | -3229.8 |  | 2213 | 131 | 1971 | 2485 |  | 2702 | 161 | 2405 | 3036 |
| ɸ(a)p(e)b(.)N(.) | 6 | 3302.3 | 490.8 | 0.0 | -3184.2 |  | 2001 | 129 | 1764 | 2270 |  | 2443 | 158 | 2152 | 2774 |
| ɸ(a+t)p(s)b(s)N(.) | 29 | 3304.1 | 492.6 | 0.0 | -3229.9 |  | 2190 | 123 | 1963 | 2444 |  | 2674 | 151 | 2395 | 2987 |
| ɸ(a)p(s)b(.)N(.) | 6 | 3308.9 | 497.5 | 0.0 | -3177.6 |  | 1954 | 128 | 1719 | 2222 |  | 2386 | 157 | 2097 | 2715 |
| ɸ(a)p(.)b(.)N(.) | 5 | 3310.2 | 498.8 | 0.0 | -3174.3 |  | 1944 | 111 | 1739 | 2174 |  | 2374 | 136 | 2121 | 2656 |
| ɸ(a+t)p(e)b(.)N(.) | 28 | 3312.4 | 501.0 | 0.0 | -3219.4 |  | 2126 | 120 | 1904 | 2373 |  | 2596 | 147 | 2323 | 2900 |
| ɸ(a+t)p(s)b(.)N(.) | 28 | 3317.3 | 505.8 | 0.0 | -3214.6 |  | 2091 | 113 | 1881 | 2324 |  | 2553 | 139 | 2295 | 2840 |
| ɸ(a+t)p(.)b(.)N(.) | 27 | 3318.5 | 507.1 | 0.0 | -3211.2 |  | 2114 | 114 | 1903 | 2348 |  | 2581 | 140 | 2321 | 2870 |

^1^ The parameters used to build these models are: survival probability (*ɸ*); capture probability (*p*); entry probability (*b*); population size (N). Each parameter may be designated as age class dependent (*a*), time dependent (*t*), constant over time (*.*) and seasonal (*s*). Full table available on S2 table.
